# Esthetic comparison of two composites used for Invisalign’s attachments bonding

**DOI:** 10.4317/jced.61853

**Published:** 2024-08-01

**Authors:** Hugo Patural, Iván Nieto-Sánchez, Cecilia Rossi, Laura Templier, Patricia Martin-Palomino-Sahagún, Inés Díaz-Renovales

**Affiliations:** 1 Postgraduate Student in Orthodontics. Alfonso X el Sabio University; 2 Part-time lecturer of the Orthodontic Postgraduate Program at University Francisco de Vitoria (UFV)- Hospital Universitario San Rafael. Madrid (Spain). (Former lecturer at UAX); 3 Private Practice

## Abstract

**Background:**

Esthetics is a factor of great importance for most patients undergoing treatment with Invisalign® aligners. When choosing a resin composite for the bonding of attachments required for the treatment, ideal characteristics such as low visibility and stain resistance of the material are essential to increase the overall perceived esthetics of the treatment. The objective of this article was to evaluate the esthetics of two resin composites used for attachment reproduction: the Transbond XT® from 3M® and the Tetric EvoCeram® from Ivoclar Vivadent®.

**Material and Methods:**

An analytic, observational, longitudinal, and prospective study was done. Attachments were bonded on 51 patients, and a dental survey about esthetics was given 3 months after the bonding of attachments. The Student’s t-test and 1-factor ANOVA tests were used, when the variables were normal, and the nonparametric alternative of the tests was used when they were not distributed normally.

**Results:**

The results indicate that there are no statistically significant differences (p>.05) in the esthetic perception of attachments according to the type of composite used.

**Conclusions:**

Even if some differences are observed between the resins, both Transbond XT® and Tetric EvoCeram® can be considered for attachments bonding from an esthetical point of view.

** Key words:**Clear aligner therapy/Dental bonding/Orthodontic appliance/Dental Esthetics.

## Introduction

When patients want or require orthodontics, they look for a fast treatment with esthetic devices. This is especially the case for adult patients whose demand for orthodontic treatments keeps increasing and who often refuse treatment with highly visible devices (1). Therapeutic alternatives such as ceramic brackets or lingual orthodontics were developed, and more recently, the thermoformed aligners, such as the ones of the brand Align Technology® (2-5), have been developed.

Invisalign® can be considered as one of the main developers (6) of this type of treatment that works by the successive change of aligners, in a modelized sequence plan (ClinCheck®) and with the help of auxiliary elements (SmartForce Features®) (7,8).

The key elements of these SmartForce Features® are the attachments, which are composite buttons bonded to the surface of the teeth (7). They must combine esthetic properties to increase the overall perception of the treatment and mechanical properties to ensure the biomechanics of the teeth movement (9). This study aimed to compare the esthetics of two resin composites used to produce these attachments.

To understand the importance of esthetics of attachments, it is essential to understand the patient profiling of people undergoing Invisalign® treatment.

According to Meier *et al*. (10), who wrote about the importance of developing esthetically superior treatment techniques, 62% of the patients (mostly adults) refused the treatment with visible devices. They concluded that Invisalign® patients were mainly women aged between 20 and 29 years and attending the clinic with high esthetic demand for both the correction of the malocclusion and the technique used to correct it. That is why developing highly esthetic aligners and attachments is paramount.

When choosing a composite for attachments reproduction, the mechanical properties and esthetical properties of the resin must be balanced (9).

Regarding the esthetical characteristics, the 2 factors of great importance are the translucency of the material and the stain resistance of it (11). An adequate translucency is required to blend with the underlying tooth. The more translucent the material is, the more the material seems to disappear over the tooth surface and the more the composite is a superior esthetic choice for the Invisalign® attachments. About the color stability, the ideal composite should have the highest resistance to color changes over time and against staining substances. The esthetical characteristics depend on material composition (resin) of the attachments. A resin composite is a material made of an organic resin matrix associated to an inorganic part thanks to a coupling agent (silane) (12-15).

This study tries to analyze the differences in esthetic perception of two different resin composites for attachments during aligner orthodontic treatment.

## Material and Methods

This study obtained the approval of the ethics committee of the Alfonso X University (2022_3/139), and the patients signed an informed consent, allowing the data to be used for scientific purpose.

This prospective, longitudinal, analytical, and observational study aims to compare the esthetic perception of two resin composites used for the bonding of attachments: the Transbond XT® (3M Unitek®) and the Tetric EvoCeram® (Ivoclar Vivadent®) with a dental survey.

**Figure 1:**
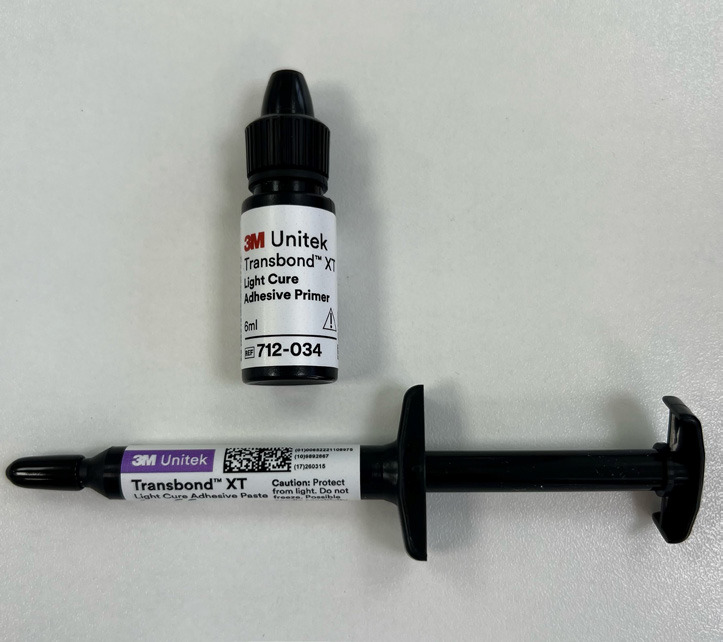
Transbond XT®.

The Transbond XT® (Fig. 1) from 3M® is a light-cured composite initially developed for orthodontic purposes and bonding of brackets. It has been used and referred in the scientific literature for the bonding of attachments (16,17). Its composition mostly comprises silane treated quartz (70-80%), BISGMA (10–20%), and bisphenol A dimethacrylate (5-10%) (18).

**Figure 2:**
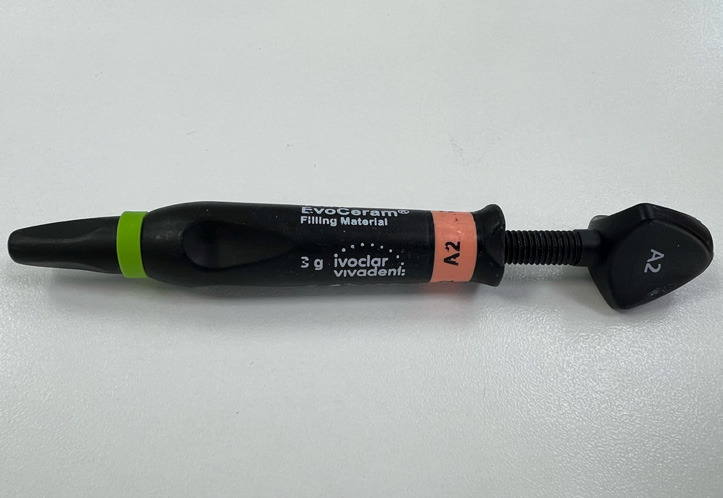
Tetric EvoCeram®.

The Tetric EvoCeram® (Fig. 2) from Ivoclar Vivadent® is a light-cured condensable composite initially developed for tooth restoration in dentistry. It has been used and referred in the scientific literature for the bonding of attachments (19). It is made of a monomer matrix of dimethacrylates (17-18%), fillers (82-83%), and additives, initiators, stabilizers, and pigments (<1%) (20).

Attachments were bonded with Transbond XT® Light Cure Adhesive Primer (3M Unitek®). The color selection was made only for Tetric Evo Ceram® (available in A2, A3, and A3.5). Transbond® is available in a single shade.

A questionnaire with 5 questions was given to the patients 3 months after the start of the treatment (T1). Participants were asked if the treatment with aligners was chosen for esthetical reasons or not, if the attachments were noticeable or not, and if color changes of attachments were observed or not and from a scale from 1 to 10, how the appearance and color of attachments were evaluated. The questionnaire was written in Spanish for the patients of the Alfonso X University.

Sample size was calculated, bearing in mind that there are 58 new aligner patients per year. This means 51 or more measurements/surveys are needed to have a confidence level of 95% so that the real value is within ±5% of the measured/surveyed value. The final sample size included 51 patients, 26 for the experimental group with Tetric Evo Ceram® and 25 for the control group with Transbond XT®.

Inclusion criteria were patients in permanent dentition between 12 and 75 years of age of both sexes, who were collaborative and gave informed consent. Patients in mixed dentition, with syndromes, undergoing orthognathic surgery and teeth with porcelain crown or composite restoration around attachments were excluded from this study.

The patients were divided into the 2 groups randomly by using a software called “Research Randomizer” available on the Internet at the following link: https://www.randomizer.org/ (21).

-Analysis of the study

The statistical analysis was carried out using the computer application IBM-SPSS Statistics version 25 (reference: IBM Corp. Released 2017. IBM SPSS Statistics v 25.0 for Windows; Armonk. NY. USA).

The descriptive analysis of the data was carried out using:

- For the contrast between means of groups of different subjects (independent of each other), the Student’s t-test and 1-factor Anova tests were used, when the variables were normal, and the nonparametric alternative of the Median Test was used when they were not distributed normally.

- The chi-square test of independence was used to cross between two categorical variables.

In all these inferential statistical tests, significance is considered when *p*<.05 (n.c. 5% usual) and high significance when *p*<.01 (n.c. 1%). It can be considered near significance, or trend toward it, when *p*<.10 (<10%).

## Results

-Description of the collected sample

The data of 51 patients from the Alfonso X University have been recorded, divided into two groups in which two types of composites have been used for the placement of attachments in orthodontic treatments with the Invisalign® technique: Tetric Evoceram® (n=26) and Transbond XT® (n=25).

The ages of these 51 patients range from 12 to 61 years (median 35) with a mean age of 37.7 years (95% CI: 33.5 - 41.9; standard deviation: ±14.9 years). A greater presence of women compared to that of men has been observed: 56.9% versus 43.1%.

-Esthetics according to composite used

This study includes a small questionnaire with 5 questions (dichotomous or on a Likert scale with a score between 0 and 10).  Table 1 shows that patients were moderately happy about the attachments’ appearance.

The answers to these questions were compared between the 2 groups established according to the type of composite.

The results ( Table 2) indicate that there are no statistically significant differences (*p*>.05) in the esthetic perception of these attachments according to the type of composite used. Only in the question regarding the color change of the attachments, a certain difference is seen, since this change was perceived more in the group with Transbond® than in the group with Tetric Evoceram® although not statistically significant (*p*>0.05) (40% vs. 19.2%).

-Age and gender

The answers were contrasted according to sex and age regardless of the group of composite used, since the previous results did not detect any difference between them.

The results of the contrast based on gender ( Table 3) indicate that no differences between men and women have been found that reach statistical significance (*p*>.05).

 Table 4 shows that age group does not affect the perceived esthetic of attachments (*p*>0.05).

## Discussion

-Translucency

In this study, the esthetics of two composites were analyzed through a survey of 5 questions focused on the following points: the reason of the aligner selection (for esthetic purposes or not), the perceived esthetics (color and general esthetics), and the attachment’s visibility and color stability.

Through the scientific literature, a similar investigation published in 2015 by Feineberg *et al*. (11) compared the same resins. It consisted of the analysis of the translucency, the stain resistance, and the hardness of 5 different composites used for Invisalign® attachments (2 of them were dental restorative composites including the Tetric EvoCeram® of our study, and 3 were orthodontic resins including the Transbond XT® from our study). In their study, the translucency and stain resistance of a resin were considered as the main factors of the esthetics of attachments. An adequate translucency was required to blend with the underlying tooth. The Tetric EvoCeram® showed higher translucency than the Transbond XT® but with the T shade (T for translucent) where the shades used in our study are opaque shades (A2, A3, and A3.5) selected according to patient teeth color to have a homogenous result between teeth and attachments. In the present study, similar results were found in terms of perceived esthetic between both composites with a slightly better appearance with the Tetric EvoCeram® without reaching statistical significance. This could explain why Tetric Evoceram® is directly recommended by Invisalign for its esthetical characteristics, while Transbond XT® was at first used for bonding brackets, without esthetic needs.

Further studies are needed to determine if esthetics is better with an opaque composite with shade selection made by patient and/or operator or with a more translucent material.

The bonding of attachment by students could affect the results of this study. Also, this study did not consider the staining habits of the patients, which could affect the esthetics of the attachments. Staining habits such as drinking red wine and coffee or smoking could affect the color stability of the attachments (22-24). This work might also be affected by the adhesive used.

According to Thai *et al*. (25), who analyzed the esthetic perception of clear aligner therapy attachments using eye-tracking technology, there is a desire for aligners with minimal attachments and ceramic brackets over clear aligners with multiple attachments. Resin composites used and their associated translucency could have affected the visibility of the attachments. These findings agree with the study of Försch *et al*. (26) who found a longer fixation time on the mouth area with aligners and attachments compared to ceramic brackets, but without observing statistically significant differences.

-Stain resistance

Another parameter studied by Feineberg *et al*. (11) was the stain resistance of the different materials. They demonstrated that both a dental restorative composite such as Tetric EvoCeram® and an orthodontic composite such as Transbond XT® were prone to staining when exposed to staining solutions, but they found a reduced stain resistance of Transbond XT® compared to Tetric EvoCeram®, and so a less esthetic appearance over time with Transbond XT®.

In our study, patients observed an important difference between the two resins, nearing statistical significance. 40% of the patients undergoing the treatment with Transbond XT® observed a change in the color of the attachments, while only 19.2% of patients for whom Tetric EvoCeram® was used observed a noticeable change in the color. These findings agree with those of the previously mentioned study (11) This difference can be explained by the initial development of these two composites, where Tetric EvoCeram® was firstly made for esthetic dental restorations, while Transbond XT® was developed for orthodontic use.

About the stain resistance, a study by Paravina *et al*. (27) studied the perceptibility threshold and acceptability threshold of the color evolution of dental ceramic. This index was applied to the work of Feinberg (11) concluding that Transbond XT® exceeded the acceptability threshold that corresponds to the limit of accepTable color differences. On the other hand, Tetric EvoCeram® only exceeded the perceptibility threshold that corresponds to a noticeable color difference. However, through a direct observation of the material, authors considered the staining of materials to be minimally visible. Further studies are required to determine the exact stain resistance of these 2 composites and more generally of composites used for attachment reproduction.

The questionnaire was given 3 months after the bonding of attachments even if according to Yap *et al*. (22), most of the staining occurred within the first week. The reason for this is that the authors wanted to let more time pass, so there were more opportunities to receive feedback from colleagues regarding attachments.

This study did not consider the habits of the patients possibly affecting the bonding success as well as the esthetics of the attachments. In 2022, Chami *et al*. (23) studied the color stability of resin composites for orthodontic attachments and referred the stain susceptibility of Transbond XT® when staining substances such as coffee and red wine are consumed, thus affecting the esthetics of the treatment. Similar studies report the color stability of Tetric EvoCeram® to staining substances. A study of Yu *et al*. (28) about the *in vitro* staining of resin composites by liquids ingested by children compared the staining of 3 different composites including Tetric EvoCeram® by different substances and concluded that Tetric EvoCeram® might have a greater stain resistance compared to other materials. This finding could justify its use for Invisalign® attachments when high esthetic is required.

-Gender and age

No significant differences were observed in this study in terms of the perceived esthetics of the two composites depending on the gender. However, a higher percentage of female chose the aligner treatment for esthetic reasons, and they were generally less satisfied by the esthetics of the attachments. From 1 to 10, they scored the color of attachment as 7.10, which males scored 7.95, and they evaluated the esthetical appearance as 6.41, which males scored 7.05. This could indicate a higher demand for esthetics in women. This agrees with Meier *et al*. (10) who found that patients interested in Invisalign® treatment were mainly women between 20 and 29 years of age interested in orthodontic treatment for esthetical reasons and rejecting visible appliances such as bonded brackets. These findings disagree with the study of Kuhlman *et al*. (29) who observed that patients considered bonded esthetic brackets more attractive than treatments with aligners as well as with the study of Livas *et al*. (30) who found no association in the perceived esthetics of clear aligner therapy between genders.

Our study did not detect significant differences in the perceived esthetics of both resins depending on the age. The group of under-30-years-old patients had the best perceived esthetics but the highest rate of perceived color changes of the attachments. Further studies are required to determine the factors impacting the perceived esthetics depending on a patient’s age.

This study is one of the first to analyze the perceived esthetics of attachments in aligner treatment. Further research should focus on other attachment materials and types of aligner used on esthetics.

## Conclusions

- From an esthetic point of view, no significant differences were observed between the two composites. Both can be used for the bonding of attachments.

- Close to near statistical significance was observed; the Transbond XT® seemed to have a reduced stain resistance as compared to the Tetric EvoCeram®, which was perceived as more esthetic by patients of this study.

- No statistically significant differences were observed in the perceived esthetics depending on the gender, but women were generally less satisfied.

- No statistically significant differences were observed in the perceived esthetics depending on age.

## Figures and Tables

**Table 1:** Perceived esthetics in the whole sample (N=51).

Questions	Composite group
	Whole sample (n=51)
1.- Choice for esthetic reasons	70.6 %
2.- Assessment of the color of the attachments	7.50 (±1.9)
3.- Esthetic appearance	6.70 (±2.00)
4.- Comments received by their attachments	33.3 %
5.- Perceive a change of color in the attachments	29.4 %

**Table 2:** Perceived esthetics. Comparison based on the composite used. (N=51).

Questions	Composite group		Contrast test	
	Tetric Evoceram (n=26)	Transbond XT (n=25)	Statistic	P-value
1.- Choice for esthetic reasons	65.4 %	76.0 %	0.69 (NS)	.406
2.- Assessment of the color of the attachments	7.58 (±1.50)	7.36 (±2.20)	0.41 (NS)	.681
3.- Esthetic appearance	6.77 (±2.05)	6.60 (±1.96)	0.30 (NS)	.764
4.- Comments received by their attachments	30.8 %	36.0 %	0.16 (NS)	.692
5.- Perceive a change of color in the attachments	19.2 %	40.0 %	2.65 (NS)	.104

(NS)= NOT significant

**Table 3:** Perceived esthetics. Comparison based on gender. (N=51).

Questions	Sex		Contrast test	
	Men(n=22)	Women (n=29)	Statistic	P-value
1.- Choice for esthetic reasons	63.6 %	75.9 %	0.90 (NS)	.343
2.- Assessment of the color of the attachments	7.95 (±1.50)	7.10 (±2.04)	1.65 (NS)	.106
3.- Esthetic appearance	7.05 (±1.99)	6.41 (±1.97)	1.13 (NS)	.265
4.- Comments received by their attachments	40.9 %	27.6 %	1.00 (NS)	.318
5.- Perceive a change of color in the attachments	31.8 %	27.6 %	0.10 (NS)	.743

(NS)= NO significant

**Table 4:** Perceived esthetics. Comparison based on the AGE. (N=51).

Questions	Age				Contrast test	
	<=30 years (n=20)	31-40 years (n=9)	41-50 years (n=9)	>=51 years (n=13)	Statistic	P-value
1.- Choice for esthetic reasons	70.0 %	77.8 %	77.8 %	61.5 %	0.96 (NS)	.810
2.- Assessment of the color of the attachments	7.80 (±1.64)	6.89 (±2.67)	7.56 (±1.13)	7.31 (±2.02)	0.53 (NS)	.666
3.- Esthetic appearance	7.05 (±2.01)	6.33 (±2.12)	6.67 (±1.22)	6.38 (±2.36)	0.40 (NS)	.751
4.- Comments received by their attachments	25.0 %	55.6 %	44.4 %	23.1 %	3.74 (NS)	.291
5.- Perceive a change of color in the attachments	35.0 %	22.2 %	33.3 %	23.1 %	0.84 (NS)	.839

(NS)= NO significant

## Data Availability

The datasets used and/or analyzed during the current study are available from the corresponding author.
